# Biopsy sampling during self-expandable metallic stent placement in acute malignant colorectal obstruction: a narrative review

**DOI:** 10.1186/s12957-021-02122-8

**Published:** 2021-02-14

**Authors:** Sigrid Skov Bennedsgaard, Lene Hjerrild Iversen

**Affiliations:** Department of Surgery, Aarhus University Hospital, Aarhus, Denmark

**Keywords:** Self-expandable metallic stent, SEMS, Colorectal stenting, Endoscopy, Colorectal cancer, Biopsy, Obstruction, Guidelines

## Abstract

**Background:**

Histopathology is a crucial part of diagnosis and treatment guidance of colorectal cancer. In Denmark, it is not routine to biopsy during self-expandable metallic stent (SEMS) placement as a treatment option for acute colorectal obstruction of unknown etiology. This is due to lack of knowledge about the risks of hemorrhage, and thus the risk to aggravate the deteriorating overview conditions. Therefore, the aim of this study is to investigate whether there is evidence to avoid biopsy sampling during acute SEMS placement.

**Methods:**

The PubMed, Embase, and Cochrane Library databases were searched for relevant studies. Studies were included if they described biopsy sampling in relation to SEMS placement. Additionally, national and international guidelines were scrutinized on Google and by visiting the websites of national and international gastrointestinal societies.

**Results:**

In total, 43 studies were included in the review. Among these, one recommended biopsy during SEMS placement, three advised against biopsy, 23 just reported biopsy was performed during the procedure, and 16 reported biopsy before or after the procedure, or the timing was not specified. Among the 12 included guidelines, only two described biopsy during SEMS placement.

**Conclusion:**

The literature on the subject is limited. In 24 of the 43 included studies, biopsy sampling was done during SEMS placement without reporting a decrease in the technical success rate. The included guidelines were characterized by a general lack of description of whether biopsy during SEMS placement should be performed or not. Prospective studies are needed in order to establish the real risk of hemorrhage, if any, when a biopsy is obtained.

**Supplementary Information:**

The online version contains supplementary material available at 10.1186/s12957-021-02122-8.

## Background

Colorectal cancer is the third most common type of cancer in the world and with high incidence rates in Europe, Australia/New Zealand, Northern America, and Eastern Asia [1]. Despite increased screening efforts, around 14% of Danish colorectal cancer patients present with a colorectal obstruction which necessitates acute care [2].

At the onset of acute colorectal obstruction, patients are often fluid and electrolyte deranged [3]. Acute bowel resection is associated with a significantly increased postoperative mortality. Thus, a diverting procedure as bridge-to-surgery can be favorable. This can be either a diverting stoma or placement of a self-expandable metallic stents (SEMS) [4]. Patients presenting with acute obstruction due to colorectal cancer more often have distant metastases than colorectal cancer patients who are diagnosed in an elective setting [4, 5]. In case of distant metastases, SEMS placement might be the definitive procedure, similarly, if patients turn out to be unfit for bowel resection [5].

Nowadays, the SEMS placement procedure in the great majority is done by inserting a flexible metal grid tube over a guide through the obstruction via an endoscope. The technical success rate of the SEMS placement is about 86% [6] and naturally depends of a clear visual field in the lumen of the colon. The procedure is also affected by the location of the malignancy, since sharply angulated or proximal anatomic locations can make it technically difficult [7]. SEMS-related complications can be perforation, stent migration, re-obstruction, bleeding, and pain [8]. An essential benefit is that the SEMS placement gives an immediate relief of obstruction and gives the possibility for the patient to be examined, get normalized fluid and electrolytes balance, become optimized of comorbidities, and undergo elective surgery [3, 5].

In order to be able to manage the patient optimally, it is necessary to know the histological diagnosis. For the great majority of patients not presenting with an acute obstruction, a biopsy is usually obtained during the elective diagnostic colonoscopy [9]. In Denmark, it has been the standard at most centers to refrain from biopsy of the tumor during SEMS placement in the acute setting, in order to avoid aggravating the already poor overview conditions with also hemorrhage—and thus possibly lose the opportunity to place the SEMS, i.e., technical failure of SEMS placement [2].

Nonetheless, if SEMS is the definitive treatment, a biopsy is needed to ensure that it is a malignant tumor as well as the histological type [10]. If no biopsy is taken from the tumor during the SEMS placement procedure, it is later on necessary to do biopsy sampling. This prolongs the process of investigation, which is not conducive for the patient.

The Danish guidelines for the diagnosis and treatment of colorectal cancer have been prepared by the Danish Colorectal Cancer Group (DCCG) [2]. The issue of biopsy sampling while placing the SEMS as a treatment for acute malignant obstruction has not been addressed in the Danish guidelines. The aim of the present study was to conduct a narrative review to investigate whether there is evidence to avoid biopsy sampling during acute SEMS placement. Further, we searched international guidelines to scrutinize their recommendations for biopsy sampling during acute SEMS placement.

## Methods

This narrative review was performed according to the PRISMA Statement [11] as appropriate.

### Eligibility criteria

We considered studies focusing on the procedure of SEMS placement in which biopsy sampling was addressed. A restriction was placed on the year of publication, excluding publications earlier than 1990. The language was set to English only. In the Embase database, publication types were restricted to article, review, and article in press.

Studies were excluded if the study was (1) not about colorectal cancer, (2) not about SEMS placement, (3) not using a standard SEMS (e.g., biodegradable stent), (4) not using colonoscopy, (5) not performed in an acute setting, or there was (6) no access to the full text publication via the institution of Aarhus University.

### Information sources and search

The PubMed and Embase databases were used to find studies focusing on the procedure of acute SEMS placement. The literature searches in the two databases were conducted on November 28 and December 3, 2020, respectively, by using four search strings (Additional file 1) for a more thorough search. Furthermore, Cochrane Library was searched for reviews regarding acute SEMS placement on December 7, 2020 (Additional file 1).

### Study selection

All the studies were transferred to Endnote™ X9, a reference management software. Then, they were transferred to the website www.covidence.org (Covidence), where the screening was done. This is an online software that can be used to organize and improve the efficiency of the screening. First, Covidence was used for the title and abstract screening and then the full text screening.

Studies identified by the searches were screened, assessed, and included according to the PRISMA statement and the Covidence directions [37]. The studies were screened by title and abstract based on the eligibility criteria.

The remaining studies went through full text screening. They were reviewed in order to assess whether they described biopsy sampling or not. It was quickly discovered that if the studies described biopsy sampling, it was only with a few words. Therefore, the search function in the PDF file was used to search for the following words, one at a time: “biops*”, “needle”, “histolog*”, “sample”, “tissue”, and “patholog*”. If the word was to be found in the study, it would be highlighted. This was the most efficient way to do the full text screening. Those that did not describe biopsy sampling at all were excluded. Studies that described biopsy sampling, but not during SEMS placement, were categorized as “before,” “after,” or “N/A” and then included. The category “before” means that the biopsy was obtained at a separate procedure, prior to the SEMS placement. The category “after” means that the biopsy was obtained at a separate procedure, after the SEMS placement was done. The category “N/A” means that the timing of the biopsy was not specified in the study.

Since the literature on the subject was so sparse, we chose to include reviews and case reports.

### Additional search: guidelines

To scrutinize guidelines on management of acute colorectal obstruction, we decided to include guidelines from countries close to Denmark (Sweden, Norway, Germany, and The Netherlands) in order to limit the number of included guidelines. Next, we included guidelines from the same countries as where the included studies originated. Thereby, it was possible to evaluate whether there was a relation between what was stated in the guidelines and what was done in practice in the studies.

To find guidelines from the selected countries and international gastrointestinal societies, it was necessary to search on Google. The words “colorectal cancer”, “gastroenterology”, “society”, and “guidelines” were used in combination with the name of the country. Other relevant societies were found on International Society Listings on www.esge.com, www.worldgastroenterology.org, and www.fascrs.com.

The guidelines were transferred to Endnote™ X9. Each guideline was reviewed in order to find out whether SEMS placement was described and if it was recommended. If so, the aim was to determine whether the guideline recommended biopsy sampling during acute SEMS placement. If the guideline recommended or did not recommend biopsy sampling, then the level of evidence was assessed using the Oxford Centre for Evidence-Based Medicine–Levels of Evidence [38].

## Results

### Study selection

**Fig. 1:**
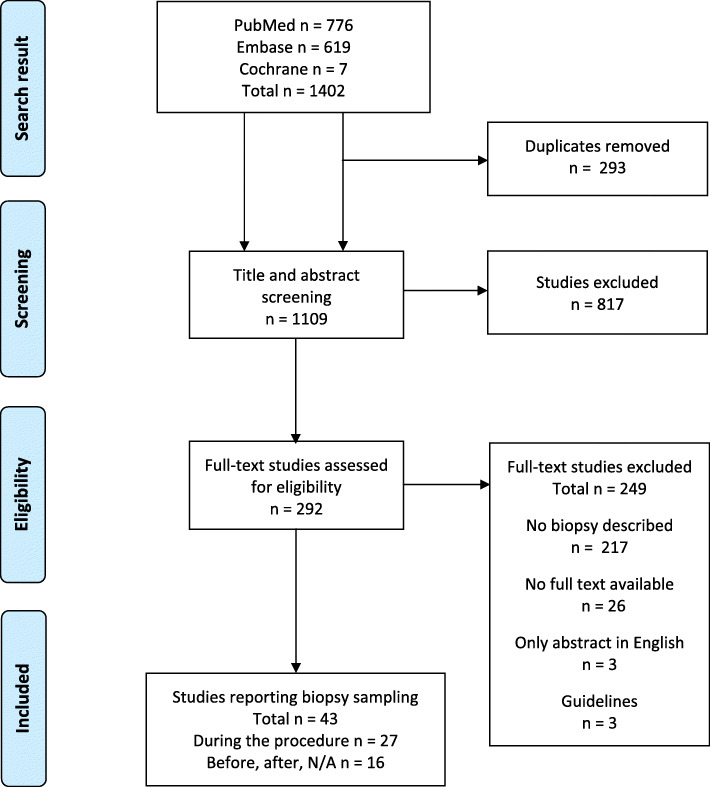
Flow chart showing the screening, assessment, and inclusion of the studies according to the PRISMA Statement [11]

Searches 1 and 2 (PubMed) yielded 776 studies (Fig. 1). Of these, 75 duplicates were removed, and 458 studies were excluded based on at least one of the exclusion criteria. Then, the remaining 243 studies went through full text screening, among which 19 studies were excluded because there was no access to the full text via the institution of Aarhus University. Besides that, 186 were excluded because they did not mention biopsy sampling. In these searches, 25 studies described biopsy sampling during SEMS placement, and 13 studies mentioned biopsy sampling before or after SEMS placement, or the time was not specified. These 38 studies were included in the study.

Searches 3 and 4 (Embase) yielded 619 studies (Fig. 1). A total of 218 duplicates were found by using Covidence and were excluded before the screening was initiated. Of the remaining 401 studies, 354 were excluded based on at least one of the exclusion criteria. During the full text screening, three guidelines were excluded, since they were outdated. Ten studies were excluded because there was no full text available, and 29 were excluded because they did not mention biopsy sampling. Two studies described biopsy sampling during SEMS placement, and three studies did not specify the timing of the biopsy. These five studies were included in the study.

The Cochrane Library search yielded 7 reviews. Five were excluded during the title and abstract screening. The remaining two reviews were excluded in the full text screening, since they did not mention biopsy sampling.

In total, the searches yielded 43 studies that described biopsy sampling: 27 reported biopsy during SEMS placement, and 16 reported biopsy before or after the procedure or not specified (Fig. 1).

### Study characteristics

The literature searches yielded 20 retrospective and ten prospective cohort studies, eight reviews, five case reports, no cross sectional studies, and no randomized studies.

Despite that several studies addressed SEMS placement, both as bridge-to-surgery and as palliation, a description of biopsy sampling during the procedure was seldom included. In total, 27 studies that described biopsy sampling during SEMS placement were included in the study (Table 1). The number of patients was rather low in the majority of studies.

**Table 1:** Biopsy during SEMS placement procedure

First author	Country	Year	Study type	***n***	Type of endoscopist	Location of malignancy	Recommends biopsy
Alshammari [12]	Saudi Arabia	2020	CR	1	GE	Left colon	N/A
Berselli [13]	Italy	2019	RS	33	GE	Left colon, splenic, or hepatic flexure	N/A
Bonfante [14]	Italy	2012	RS	45	S	Left colon, rectum	N/A
Donlon [15]	Ireland	2019	RS	103	N/A	All locations	N/A
Dulucq [16]	France	2006	PS	14	GE	Left colon, right colon, rectum	N/A
Feo [17]	USA	2011	R	-	N/A	Left colon, right colon	N/A
Fernández-Esparrach [18]	Spain	2010	RS	39	GE	Left colon, rectum	N/A
Gianotti [19]	Italy	2013	PS	83	GE	Left colon, right colon	N/A
Han [20]	China	2020	RS	14	S	Left colon, splenic flexure	N/A
Jost [21]	Switzerland	2007	RS	67	N/A	Left colon, splenic flexure, rectum	Yes
Keymling [39]	Germany	2003	R	-	N/A	All locations	N/A
Kuwai [22]	Japan	2018	PS	501	N/A	Left colon, right colon	N/A
Könes [23]	Turkey	2019	RS	21	GS	Splenic or hepatic flexure	N/A
Lee [24]	South Korea	2012	RS	88	GE	Left colon, right colon, rectum	N/A
Li [40]	China	2019	RS	26	GE	Left colon, splenic flexure, rectum	N/A
Liu [25]	China	2019	CR	6	CS	Left colon, rectum	N/A
Matsuda [26]	Japan	2016	RS	28	CS	Left colon, right colon, rectum	N/A
Matsuda [27]	Japan	2019	RS	25	S	Left colon, right colon, rectum	N/A
Matsuzawa [28]	Japan	2015	PS	513	GE	All locations	No
Pacheco-Barcia [29]	Spain	2019	RS	78	N/A	Left colon, transverse colon	N/A
Repici [30]	Italy	2006	R	-	N/A	Left colon, right colon	N/A
Repici [31]	Italy	2008	PS	42	N/A	All locations	N/A
Saito [32]	Japan	2015	PS	296	N/A	Left colon, right colon, rectum	No
Soto [33]	Spain	2006	RS	62	N/A	Left colon, right colon, rectum	N/A
Stipa [34]	Italy	2007	PS	30	N/A	Left colon, splenic or hepatic flexure, rectum	N/A
Tomita [35]	Japan	2019	PS	404	N/A	Left colon, right colon, rectum	No
van den Berg [36]	The Netherlands	2014	RS	59	GE	Left colon, right colon, splenic flexure	N/A

Study type: *CR* case report, *RS* retrospective study, *PS* prospective study, *R* review

Type of endoscopist: *GE* gastroenterologist, *CS* colorectal surgeon, *GS* general surgeon, *S* surgeon (not specified)

The included studies were from 17 different countries. Three studies [28, 32, 35], all from Japan, recommended to omit biopsy during SEMS placement. One of the three studies was the prospective cohort study by Matsuzawa et al. [28]. During the study period, the research group made a website with a brief guideline [41] for SEMS placement. It was this particular guideline the three studies based their recommendations on. Another one of the three studies was Saito et al. [32], a prospective cohort study with 312 patients. The authors reported that biopsy sampling during the procedure was not recommended: “To maintain good visualization of the tumor orifice, biopsy immediately before SEMS placement was not recommended because the tumor orifice becomes obscure due to bleeding.” [32].

The opposite was stated in the study of Kuwai et al. [22]. This study was a prospective cohort study with 511 patients. The study analyzed factors related to difficult SEMS placement. There were 10 technical failures, so the analysis was done on the remaining 501 patients. One of the analyzed factors was biopsy right before placing the SEMS. The study included both a univariate and a multivariate analysis of relationships between interventional practices and technically difficult SEMS placement procedures. The procedures were categorized as technically difficult, if the procedure time exceeded 45 min. For performed biopsy sampling, the OR (95% CI) in the multivariate analysis was 1.4 (0.88–2.19), i.e., no significant difference in the technical difficulty—as measured by prolonged procedure time.

In Jost et al. [21], SEMS placement with biopsy was recommended, but the recommendation should be characterized as an unjustified opinion only: “We recommend combined endoscopic and fluoroscopic guidance for colorectal stent placement for the following reasons: (a) a biopsy of the lesion can be taken and allows initial investigation with histology…” [21].

A total of 23 studies described biopsy during the SEMS placement procedure, but did not directly recommend biopsy during the procedure (Table 1). The 23 studies were included in this review, since it was not stated that the biopsy led to technical failure or a decrease in the technical success rate. Therefore, it was interpreted as safe to biopsy during the procedure.

The 16 studies that mentioned biopsy sampling, but not during the placement procedure, were also included (Table 2). These studies were included in the study because they might be able to provide insight in the clinical consequences of this treatment strategy. The studies showed that it is not only a Danish practice to refrain from biopsy during SEMS placement.

**Table 2:** Biopsy before or after SEMS placement or time not specified

First author	Country	Year	Study type	***n***	Type of endoscopist	Location of malignancy	Recommends biopsy
**Time of biopsy: before**							
Fugazza [42]	Italy	2017	R	-	N/A	Left colon, right colon, rectum	N/A
**Time of biopsy: after**							
Adler [43]	USA	2002	CR	1	S	Rectum	N/A
Bayraktar [44]	Turkey	2015	RS	49	GS	Left colon, rectum	N/A
Larkin [45]	Ireland	2014	PS	44	CS	All locations	N/A
**Time of biopsy: N/A**							
An [46]	China	2020	RS	139	S	Left colon, rectum	N/A
Chung [47]	South Korea	2012	CR	1	N/A	Left colon	N/A
Dítě [48]	Czech Republic	2003	R	-	GE	Left colon, rectum	No
Farkas [49]	UK	2019	R	-	N/A	Left colon	N/A
Kaplan [50]	USA	2014	R	-	N/A	Left colon, right colon, rectum	N/A
Kim [51]	South Korea	2012	RS	14	N/A	N/A	N/A
Li [52]	China	2019	CR	1	N/A	Left colon	N/A
Öistämö [4]	Sweden	2016	RS	20	CS	Left colon, splenic flexure	N/A
Parodi [53]	Italy	2016	RS	88	GE	Left colon, rectum	N/A
Pisano [54]	Italy	2018	R	-	S	Left colon	Yes
White [55]	Australia	2011	RS	2	N/A	Left colon	N/A
Yanar [56]	Turkey	2014	PS	42	N/A	Left colon, rectum	N/A

Study type: *CR* case report, *RS* retrospective study, *PS* prospective study, *R* review

Type of endoscopist: *GE* gastroenterologist, *CS* colorectal surgeon, *GS* general surgeon, *S* surgeon (not specified)

Even though most of the included studies reported the location of malignancy, i.e., right colon, left colon, or rectum, none of them addressed biopsy sampling in relation to the localization.

### Additional results: guidelines

No guidelines from countries close to Denmark, i.e., Sweden, Norway, The Netherlands, nor Germany included any guidance of biopsy during SEMS placement. Guidelines from the same countries as the included studies were reviewed: Ireland, Great Britain, France, Czech Republic, Switzerland, Spain, Japan, Australia, and the USA. The European Society of Gastroenterology’s (ESGE) guideline was reviewed because three of the included studies were from European countries that used the ESGE guideline.

It was not possible to find national guidelines in English from neither Italy, Turkey, Saudi Arabia, China, nor South Korea. When searching for the guidelines from these five countries, the relevant websites were in foreign languages. This made the search challenging.

In summary, we found one Danish guideline, one international guideline, and ten different national guidelines, which were included (Table 3).

**Table 3:** Descriptions of and recommendations on SEMS in the included guidelines

Country/society	Year	Description of SEMS placement	Recommends SEMS as treatment^**a**^	Recommends biopsy^**b**^	Evidence degree for biopsy^**b**^
Denmark [2]	2019	Yes	Yes	No	-
Sweden [57]	2020	Yes	Yes	No	-
Norway [58]	2019	Yes	Yes	No	-
The Netherlands [59]	2019	Yes	Yes	No	-
Germany [60]	2019	Yes	Yes	No	-
Ireland [61]	2017	Yes	Yes	No	-
Great Britain [61]	2017	Yes	Yes	No	-
Switzerland [62]	2020	Yes	Yes	Yes	D
Spain [63, 64]	2017 2019	Yes Yes	Yes Yes	No No	- -
Czech Republic [62]	2020	Yes	Yes	Yes	D
European Society (ESGE) [62]	2020	Yes	Yes	Yes	D
American Society (ASCRS) [63, 65]	2017 2020	Yes Yes	Yes Yes	No No	- -
Japan [41]	2015	Yes	Yes	Yes	D
France [62]	2020	Yes	Yes	Yes	D
Australia [66]	2018	Yes	Yes^c^	No	-
Saudi Arabia	-	-	-	-	-
China	-	-	-	-	-
South Korea	-	-	-	-	-
Turkey	-	-	-	-	-
Italy	-	-	-	-	-

^a^Of acute colorectal obstruction

^b^During SEMS placement

^c^For patients with increased risk of postoperative mortality

Only two guidelines described biopsy sampling during SEMS placement [41, 62]. One of these was the ESGE Clinical Guideline from 2020 [62]. In the ESGE guideline, biopsy during the SEMS placement procedure was described as a preferable procedure, since histopathology results can benefit the further management of the patient. However, the ESGE guideline stated that “…pathological confirmation of malignancy should not persistently be pursued in an urgent setting, such as during stent placement for acute colonic obstruction.” In the guideline, the recommendation is categorized as a strong recommendation with low-quality evidence.

The other guideline which described biopsy sampling was made by Japan Colonic Stent Safe Procedure Research Group. The guideline stated that biopsies should “be kept to an absolute minimum in order to secure the best possible visual field”.

## Discussion

We searched the literature to investigate whether there was evidence for avoiding biopsy during SEMS placement. The majority of the studies lacked detailed descriptions on when to biopsy, and what the risks might be. This challenged the assessment of the applicability of the studies. Three studies did not recommend biopsy during the procedure, one study recommended it, and 23 studies described biopsy sampling during SEMS placement without reporting a decrease in the technical success rate. Only one study, Kuwai et al. [22] included a statistical analysis of biopsy as a factor related to difficult SEMS placement. However, biopsy was not significantly associated with technical difficulties (defined as procedure time exceeding 45 min) during the SEMS placement procedure.

These results leave one with the overall impression that biopsy can be performed safely during SEMS placement. Even so, the results were not strikingly convincing with only one study including statistical data analysis.

For the majority of studies, there was no information about when and how the surgeons got a tissue sample from the tumor. In some cases, they might have performed biopsy during the SEMS placement, but chose not to report it in the study because it was considered a normal procedure without interest to others. On the other hand, there could also be cases where they did not biopsy. Regarding to these cases, it would have been interesting to know why they did not biopsy. There might be the possibilities that biopsy during SEMS might be tried without success or be not widely known as surely safe. Information like these could improve the quality of present study and help clarify what kind of research needs to be done on the subject.

Only two guidelines described biopsy sampling [41, 62]. ESGE has a Guidelines Committee, and each guideline is made by a group of experts [67], which is considered a good quality of the guideline [62]. It should be noted that the guideline’s references (in the section of interest) aimed to study brush cytology and biopsy sensitivity in general—not specifically during SEMS placement. Thus, it remains unclear on what basis the ESGE guideline recommends biopsy during the SEMS placement procedure, and the recommendation in present aspect must be considered weak.

The other guideline that described biopsy sampling was made by a Japanese research group [41]. The guideline did not recommend biopsy during SEMS placement. However, the recommendation was without any references, except their own prospective cohort study. The guideline only explained briefly how hemorrhage during the procedure can make it difficult to identify the stenotic lumen.

Comparing the guidelines and studies from the same country showed no clear connection between what was described in the guidelines and what was done in the studies. The reason for this is uncertain. Maybe the decision on when to biopsy is usually made by the individual hospital/center. Or it might not be a high priority to include it in the guidelines.

The strengths of present study include the meticulous literature search and the systematic screening and assessment of the search results. Additionally, the study does not only assess studies but also national and international guidelines of relevance to the literature.

This review has limitations, among others, many of the studies completely lack information on when and how the biopsy was performed, and furthermore how the biopsy affects the procedure of treatment. Due to the absence of randomized trials, the study was based on retrospective and prospective cohort studies, reviews, and case reports. Therefore, it was not possible to yield any summary measures like risk ratio. Similarly, assessing risk of bias of the individual studies was challenged by the huge heterogeneity of the studies. Only one study aimed to analyse how biopsy affects SEMS placement. This made it difficult to make a proper comparison of the included studies.

Furthermore, a limitation of present study is the search for guidelines, which was difficult since there is no guideline database. Therefore, it was difficult to know for sure whether the search was adequate. In order to limit the number of guidelines, only countries close to Denmark and countries, where the included studies originated from, were included. This means that it was a selective, but well-defined review only of guidelines.

## Conclusions

We must conclude that the literature on recommendation for or against biopsy sampling during SEMS placement in acute malignant colorectal obstruction is very limited. No convincing evidence was found to avoid biopsy during SEMS placement—neither in the studies, nor the guidelines. Furthermore, this study showed that the majority of national guidelines lack recommendations on whether biopsy sampling should be performed or not during SEMS placement.

## Supplementary Information

**Additional file 1.** The search strings used in PubMed, Embase, and Cochrane Library.

## Data Availability

Data sharing is not applicable to this article as no datasets were generated or analyzed during the current study.

## Abbreviations

SEMS: Self-expandable metallic stent
ESGE: European Society of Gastroenterology
