# Supplementary material for: Biopsy sampling during self-expandable metallic stent placement in acute malignant colorectal obstruction: a narrative review
Source: World J Surg Oncol. 2021 Feb 14;19:48. doi: 10.1186/s12957-021-02122-8 (PMC7883457; doi:10.1186/s12957-021-02122-8)
Supplement: Supplementary file 1 — Additional file 1. The search strings used in PubMed, Embase, and Cochrane Library. [file 12957_2021_2122_MOESM1_ESM.docx]

**Figure 1**

| PubMed, 28^th^ November 2020 |
| --- |
| (biopsies OR biopsy OR "Biopsy"[Mesh] OR "Biopsy, Needle"[Mesh] OR pathology OR "Pathology, Surgical"[Mesh] OR histology) AND ("Colorectal Neoplasms"[Mesh] OR "colorectal neoplasm" OR "obstructive cancer" OR "colorectal obstruction" OR "colon cancer" OR "colonic neoplasm" OR "Colonic Neoplasms"[Mesh] OR "colorectal cancer" OR CRC OR "acute colorectal obstruction" OR "acute colon obstruction" OR "Intestinal Obstruction" OR "Intestinal Obstruction"[Mesh]) AND ("Self Expandable Metallic Stents" OR "Self Expandable Metallic Stents"[Mesh] OR SEMS OR stent OR stents) |
| PubMed, 28^th^ November 2020 |
| ("acute colorectal obstruction" OR "acute colon obstruction" OR "Intestinal Obstruction"[Mesh]) AND (stents OR "Self Expandable Metallic Stents"[Mesh] OR SEMS "endoscopic stenting" OR "Self Expandable Metallic Stents" OR "endoscopic treatment") |
| Embase, 3^rd^ December 2020 |
| ('biopsy'/exp OR biopsy OR 'biopsies'/exp OR biopsies OR 'histology'/exp OR histology OR 'pathology'/exp OR pathology OR 'biopsy needle'/exp OR 'colon biopsy'/exp) AND  ('colorectal neoplasm'/exp OR 'colorectal neoplasm' OR 'obstructive cancer' OR 'colorectal obstruction'/exp OR 'colorectal obstruction' OR 'colon cancer'/exp OR 'colon cancer' OR 'colonic neoplasm' OR 'colorectal cancer'/exp OR 'colorectal cancer' OR crc OR 'acute colorectal obstruction' OR 'acute colon obstruction' OR 'intestinal obstruction'/exp OR 'intestinal obstruction') AND ('self expandable metallic stents'/exp OR 'self expandable metallic stents' OR sems OR 'stent'/exp OR stent OR 'stents'/exp OR stents) |
| Embase, 3^rd^ December 2020 |
| ('acute colorectal obstruction' OR 'acute colon obstruction' OR 'intestinal obstruction'/exp OR 'intestinal obstruction' OR 'colon obstruction'/exp) AND (('stents'/exp OR stents OR 'self expandable metallic stents'/exp OR 'self expandable metallic stents' OR sems) AND ('endoscopic stenting'/exp OR 'endoscopic stenting') OR 'endoscopic treatment') |
| Cochrane, 7^th^ December 2020 |
| stents AND ’colorectal neoplasm’ AND obstruction |
